# Engaging a community to focus on upper limb function in people with multiple sclerosis: the ThinkHand campaign case study

**DOI:** 10.1186/s40900-024-00586-y

**Published:** 2024-06-18

**Authors:** Alison Thomson, Rachel Horne, Christine Chapman, Trishna Bharadia, Patrick Burke, Elizabeth Colwell, Mark Harrington, Bonnie Boskovic, Andrea Stennett, David Baker, Gavin Giovannoni, Klaus Schmierer

**Affiliations:** 1 Centre for Preventive Neurology, Wolfson Institute of Population Health, Queen Mary University of London, London, UK; 2 Patient Author, Barts MS Advisory Group, Queen Mary University of London, London, UK; 3 The Blizard Institute, Centre for Neuroscience, Surgery and Trauma, Queen Mary University of London, London, UK; 4 Clinical Board Medicine (Neuroscience), Barts Health NHS Trust, The Royal London Hospital, London, UK

**Keywords:** Multiple Sclerosis, Patient public involvement, Public engagement, Design, Clinical trials, Patient-centred, Co-creation

## Abstract

Background: Solving complex research challenges requires innovative thinking and alternative approaches to traditional methods. One such example is the problem of arm and hand, or upper limb function in multiple sclerosis (MS), a neurological condition affecting approximately 2.9 million people worldwide and more than 150,000 in the United Kingdom. Historically, clinical trials and research have focused on mobility and walking ability. This excludes a large number of patients who are wheelchair users, limiting their quality of life and restricting access to possibly helpful medications. To address this issue, the ThinkHand campaign was launched in 2016, aiming to raise awareness about the importance of upper limb function in MS and develop alternative ways to measure, record, and account for hand and arm function changes.

Main body: The campaign utilised innovative strategies at scientific conferences and online surveys to engage people affected by MS, healthcare professionals, charities, and researchers in discussing the importance of preserving upper limb function. Through co-design and interdisciplinary collaboration, the campaign developed new tools like the low-cost cardboard version of the Nine-Hole Peg Test, facilitating remote monitoring of hand function. Additionally, the campaign co-created the “Under & Over” rehabilitation tool, allowing individuals with advanced MS to participate in a remote rehabilitation program.

The impact of the ThinkHand campaign has been significant, helping to shift the focus of both academic and industry-supported trials, including the O’HAND and ChariotMS trials, both using upper limb function as their primary end point. The campaign’s patient-centred approach highlighted the importance of recognising patients’ perspectives in research and challenged established assumptions and practices. It demonstrated the effectiveness of interdisciplinary collaboration, systems thinking, and co-creation with stakeholders in tackling complex problems.

Conclusion: The ThinkHand campaign provides valuable insights for health research practices. By involving patients at all stages, researchers can gain a deeper understanding of the impact of disease on their lives, identify gaps and focus research on their needs. Experimentation and iteration can lead to innovative solutions, and openness to unconventional methods can drive widespread change. The ThinkHand campaign exemplifies the potential of patient-centred approaches to address complex research challenges and revolutionise the field of MS research and management. Embracing such approaches will contribute to more inclusive and impactful research in the future.

## Abstract (plain language summary)

Solving complex research challenges requires creative thinking and new ways of doing things. One such challenge is understanding the problems with arm and hand function in multiple sclerosis (MS), a neurological condition that affects more than 150,000 in the United Kingdom. In the past, research focused mainly on walking ability, leaving out many people who use wheelchairs.

To tackle this issue, we created the ThinkHand campaign in 2016. Its goal was to raise awareness about the importance of hand and arm function for people with MS (pwMS) and find better ways to measure changes in these functions such that they can become outcomes in clinical trials. This could provide a pathway to better treatments for pwMS who cannot walk.

The campaign used various methods, including surveys, social media posts, exhibitions and music to involve pwMS, healthcare professionals, charities, and researchers in discussions about the issues. Working together, they created tools to support pwMS, particularly those at an advanced stage of the disease (pwAMS), to take part in research and measure their hand and arm function. Through our collaborative approach focusing on patients’ perspectives, the campaign challenged old ideas and deeply embedded practices. It showed that collaboration between different areas of expertise involving pwMS at all stages of research can help solve complex problems. This campaign teaches us valuable lessons for health research. When researchers listen to patients and try new things, they can better understand how a disease affects people’s lives and develop better solutions.

In conclusion, we show how embracing a patient-centred approach can address complex research challenges and improve how we study and manage MS and other conditions in the future.

## Background

Solving complex research challenges requires innovative thinking and alternative approaches to traditional methods. One such example is the problem of arm and hand, or upper limb function, in multiple sclerosis (MS), a neurological condition affecting approximately 2.9 million people worldwide and more than 150,000 in the United Kingdom (UK) [1, 2]. The historical focus on mobility and walking ability, dominated by lower limb function, in clinical trials and research, has potentially negative implications on the quality of life of some individuals with MS, particularly in the more advanced stages. Indeed, there is also evidence to suggest that some newly diagnosed people with MS (pwMS) present with upper limb impairment which increases in severity over time [3, 4]. Yet, the focus on lower limb function has meant that, historically, the vast majority of trials have excluded patients who are wheelchair users [5]. The main reason for this understanding was that treatment would not be beneficial given the nature of the condition in these individuals since its usefulness was being measured by improvement in lower limb function [6]. This means that to date, MS disease-modifying treatments (DMTs) had only been licensed if they improve walking ability. To address this problem, our group launched the ThinkHand campaign in 2016. The key aims were set out and made public to raise awareness about the importance of upper limb function in MS, the socioeconomic costs of losing upper limb function and to develop alternative ways to measure, record, and account for the change in hand and arm function in the everyday lives of people with MS.

The early and innovative contribution of people with MS in this project and the creative methods used, enabled the ThinkHand campaign to successfully bring this “wicked problem” to the fore, while having a long-lasting impact on the field of MS research and management. “Wicked” because of its (i) bias and exclusion of a large number of pwMS from MS research participation opportunities and (ii) complexity and interrelated causes and consequences. “Wicked problems”, a term coined by Rittel and Webber [7] describes complex social problems that are difficult to solve due to their interrelated causes and consequences, the lack of a clear problem definition, the presence of multiple stakeholders with conflicting values and goals, and the unpredictable nature of their evolution. These problems often defy traditional problem-solving approaches and require novel, innovative, and collaborative solutions that address the underlying systemic issues. In the case of the ThinkHand campaign, it also required pathophysiological underpinning, which was achieved through hypothesis-generating and supporting, preliminary evidence [8–10]. The multi-faceted and unpredictable nature of MS and its impact on upper limb function makes it difficult to measure and track effectively. This issue is also interconnected with other factors such as walking ability, cognition, quality of life, employability, and daily living activities, adding to its complexity. The lack of consensus among stakeholders, including patients, healthcare providers, researchers, and policymakers, on the most effective ways to measure and track upper limb function in MS also makes it challenging to find a comprehensive solution.

The aim of this case study is to document the impact of the ThinkHand campaign and demonstrate the potential for patient-centred approaches to address complex issues in health research. The article highlights the importance of recognising that traditional methods of knowledge dissemination and mobilisation within science and academia have limited reach and impact, and that alternative methods from disciplines such as art and design can open up a topic for debate, invite people with lived experience of MS to challenge assumptions and ultimately change clinical and research practice. The article also highlights the importance of actively involving patients, their families, and members of the public in the design, conduct, and dissemination of research activities, which is now considered best practice in the UK. This can take many forms, such as patient advisory groups, patient representatives on research teams, and patient-led research projects. The goal of patient and public involvement (PPI) in research is to ensure that the needs, concerns, and perspectives of patients and the public are taken into account in the research process. ThinkHand demonstrates how through involving patients and the public, researchers can gain a deeper understanding of the impact of disease on their lives and where they want research to focus. Further, it demonstrates how engaging people living with the conditions can help tackle “wicked problems” and in turn change a field of research.

## The problem within the field

The decision to raise awareness about the importance of hand and arm function in MS stemmed from the realisation that clinical trials had shown that DMTs in individuals with advanced stages of MS can still delay the worsening of disability in hand function, despite having little to no impact on leg function. The effect on hand function was observed in trials where it had been employed as a secondary endpoint. This is a problem if the only outcome measure is around walking ability, as measured by the Expanded Disability Status Scale (EDSS) [11, 12]. This observation has been reported in neurology literature dating back to the early 1990s but has not been acted on by the MS community. In light of this, the research team at QMUL and Barts Health NHS Trust revisited a model previously – and ironically – suggested by John Kurtzke [13], the inventor of the EDSS, to describe progressive MS as a “length-dependent central nervous system axonopathy” [8]. This model suggests that the longer the nerve fibres, the more likely they are affected to a functionally relevant degree by MS lesions and, ultimately, die off. This is why progressive MS symptoms are often seen first in the legs, as the nerve fibres supplying the legs are longer than those to the arms. This concept is key to testing whether people with advanced MS using wheelchairs can still expect improvements in hand and arm function.

Through responsive and meaningful patient involvement, the ThinkHand campaign aimed to raise awareness and initiate discussions among people with MS, clinicians, charities, pharmaceutical companies, and regulators about the importance of preserving upper limb function for people with more advanced MS. The campaign outlined specific goals to impact [1] trials in more advanced MS to include wheelchair users [2], regulators to accept the Nine-Hole Peg Test (9HPT) as a primary outcome measure for phase 3 trials [3], treatments for people with more advanced MS (wheelchair users) to protect arm and hand function and [4] evidence-based stopping criteria for DMTs.

## How we addressed the issue

**Fig. 1:**
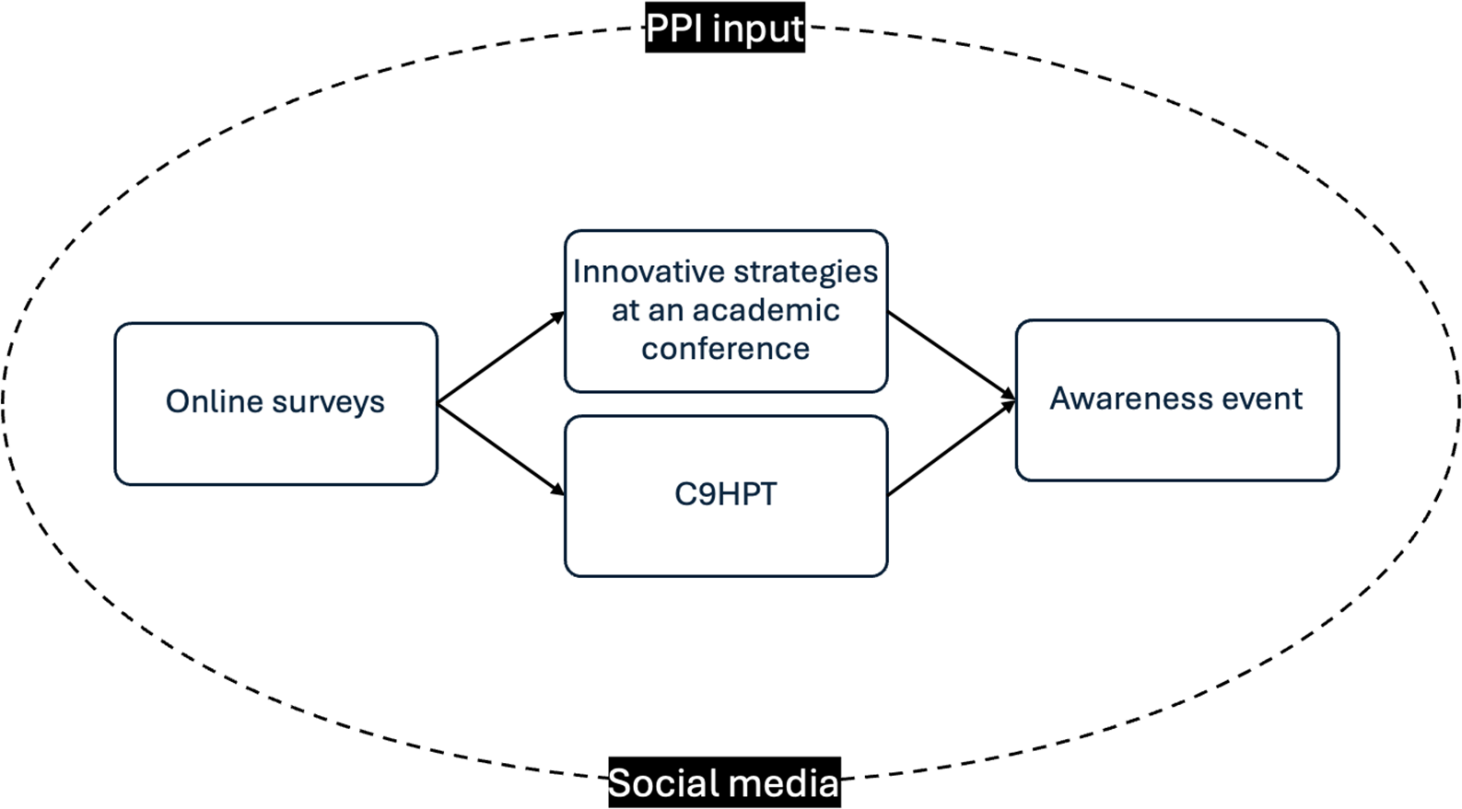
Workflow diagram of the stages of the campaign

The method used in the campaign, shown in Fig. 1, was a multi-pronged approach that involved online surveys, disruptive strategies at scientific conferences, awareness events, and co-design of outcome measurement and rehabilitation tools. The following provides further details of the methods used:

- **Online Surveys**: Online surveys were conducted through The MS Research blog [14] to gather data on the importance of hand and arm function in daily life for individuals with advanced MS and the attitudes of healthcare professionals towards including such individuals in research studies.
- **Social Media**: explanatory videos documenting the pathway to ChariotMS trial [15].
- **Innovative Strategies**: To disseminate the knowledge obtained from the online surveys and encourage discussion around the issue, unconventional activities were employed at the European Committee for Treatment and Research in Multiple Sclerosis (ECTRIMS) conference in 2016. These included researchers constructing their research poster out of cardboard using their own hands emphasising the importance of hand function and inviting people with MS to contribute to a “Burning Debate” session on the inclusion of wheelchair users in MS clinical trials.
- **Awareness Event**: The campaign organised a ThinkHand awareness event in London, which celebrated the hand function of artists, musicians, and a renowned jewellery maker, all living with MS. The aim of the event was to highlight the impact of hand and arm function on self-expression, livelihood, creativity, and independence.
- **Low-Cost Assessment Tool**: The 9HPT is an outcome measure used to measure finger dexterity and arm function and is considered the gold standard for assessing upper limb function in people with MS in clinical trials. Because the commercial apparatus, made of plastic, is quite expensive (around GBP105), we created a low-cost (GBP9.99), environmentally friendly version for people to use at home so they could monitor their arm and hand function themselves. The cardboard tool was exhibited at ECTRIMS 2017 and an academic publication detailing its validation was produced [16].
- **Co-Design of Rehabilitation Tool**: The focus on patient-generated knowledge in the campaign led to the co-design of a hand rehabilitation tool “Under & Over” with individuals living with MS. A randomised controlled study was conducted to assess the delivery of a remote rehabilitation program to individuals with advanced MS [17].

## Role of patient engagement

The MS Research group at QMUL has a long-standing PPI group and a track record of engaging people at all stages of research development [18–21]. Clinical academics and researchers within the group successfully use a range of social media platforms within their PPI and engagement work, which became crucial in this campaign. The established PPI group, along with a number of other people with MS (co-authors on this paper and listed in the acknowledgements) contributed to the formulation of how to tackle the wicked problem, contributing their personal experiences and personal insights of the issue. For example, people were sharing experiences such as, “Now that I’m in a wheelchair, my hands have become my legs.”

## Online survey

This led to the campaign initiating a series of online surveys [6]. These were conducted through The MS Research Blog, which had a daily readership of 9,000 individuals. The surveys invited people with advanced MS to share the importance of hand and arm function in their daily lives and healthcare professionals to share their attitudes towards including individuals with advanced MS in research studies. 360 people with MS and 43 MS Neurologists responded to the surveys. The findings revealed that 92% of respondents (321/349) placed greater importance on their upper limb function compared to lower limb function, and 95% (332/350) felt that pwMS using wheelchairs should not be excluded from DMT trials. At the same time, 75% of UK MS Neurologists (32/43) said that they regularly stop prescribing DMTs in pwMS who need a wheelchair, even though 61% (26/43) felt that currently available DMTs were likely still effective even at this stage of the disease. The results of these surveys focused the ThinkHand campaign efforts on changing perceptions of the problem. The team needed to go beyond traditional methods of communication and knowledge dissemination to illustrate the impact of these issues on a larger scale.

## Innovative strategies and social media

**Fig. 2:**
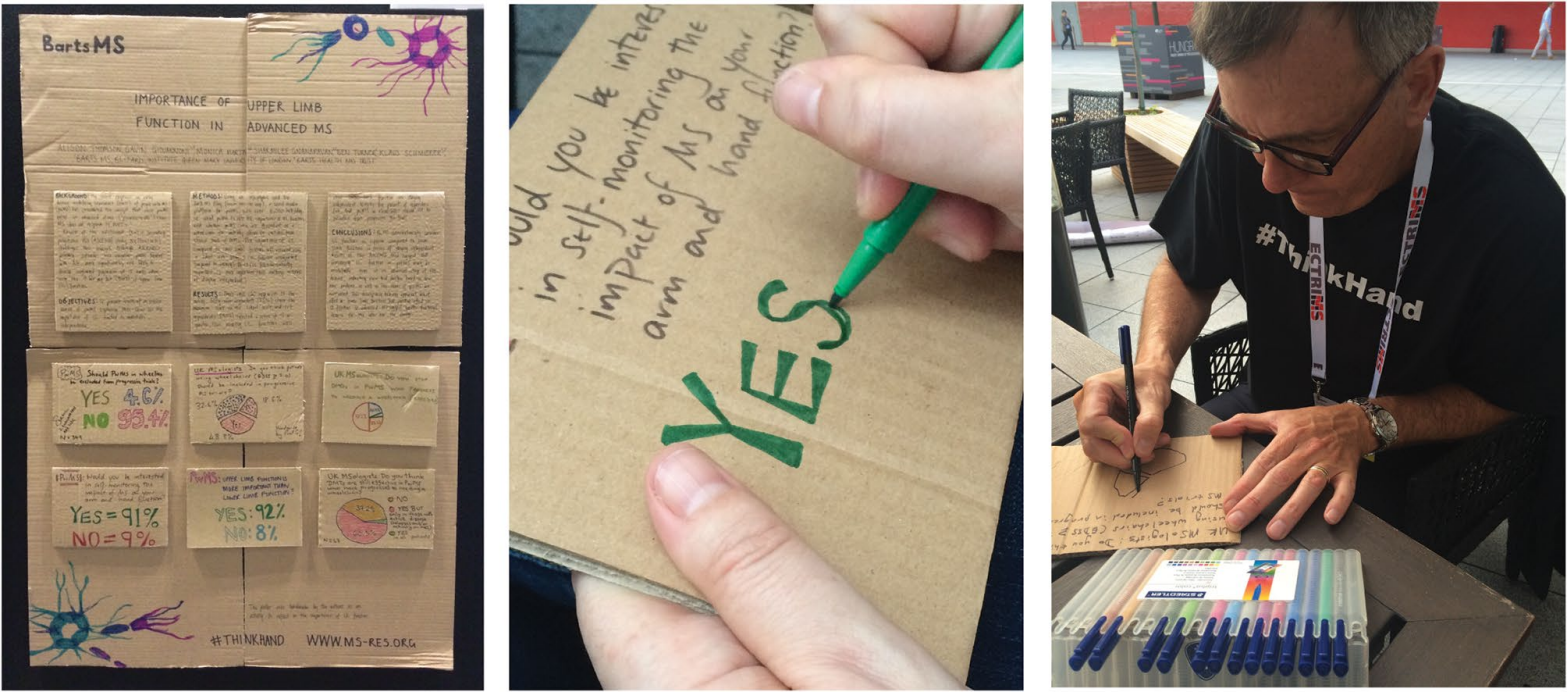
The cardboard poster presented at ECTRIMS conference 2017, being made by poster authors by hand

**Fig. 3:**
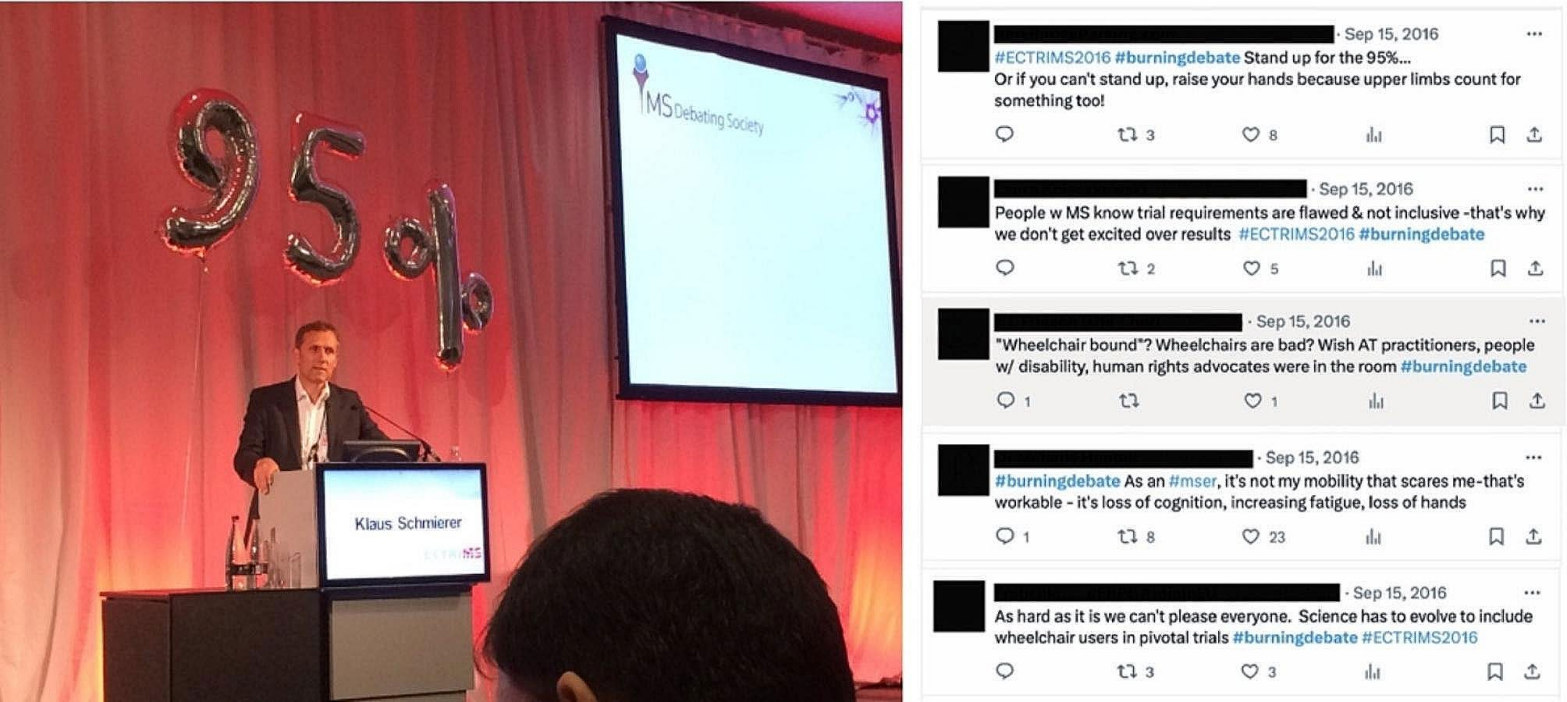
Professor Klaus Schmierer taking part in the Burning Debate and Twitter commentary from patients

To do this, they employed disruptive strategies at a clinical academic conference in 2017 to draw attention to the survey results and the wider issue. ECTRIMS is the largest worldwide conference dedicated to understanding and treating MS and is attended by thousands of MS specialist healthcare professionals, researchers, and pharmaceutical companies. The survey results were created by hand by the authors into a poster made of cardboard to emphasise the importance of hand function in daily life (Fig. 2). The team also hosted a “Burning Debate” on the inclusion of wheelchair users in clinical trials, utilising the survey data and with a backdrop of inflatable numbers (see Fig. 3) to support the argument. The balloons displayed the number “95%,” representing the data and indicating the percentage of survey respondents who believe that people with MS using wheelchairs should not be excluded from progressive trials. People with MS were invited to the conference debate through Twitter (now X), where they contributed comments on the discussion (see Fig. 3). These activities brought attention to the issue with conference participants engaging in these activities and reflecting on their own assumptions and approaches about managing and researching upper limb function for the people they research and treat. Unconventional elements such as balloons and handmade posters were used to creatively emphasise critical issues at the academic conference, disrupting traditional approaches. Additionally, involving people with MS in the debate through social media challenged assumptions and brought a fresh perspective to the discussion.

## Awareness event

The ThinkHand exhibition was crucial to raising awareness about the impact of hand and arm function on key aspects of life such as self-expression, livelihood, and creativity. The exhibition showcased work of artists, musicians, and a renowned jewellery maker who have MS. The aim was to communicate the human aspect of upper limb function and its link to one’s identity. It was held in London on February 22nd 2018 and engaged funders, regulators, and researchers to reflect on this issue. It was covered by the Evening Standard [22] and various multimedia channels within the MS community [23–26].

## Low-cost assessment tool

**Fig. 4:**
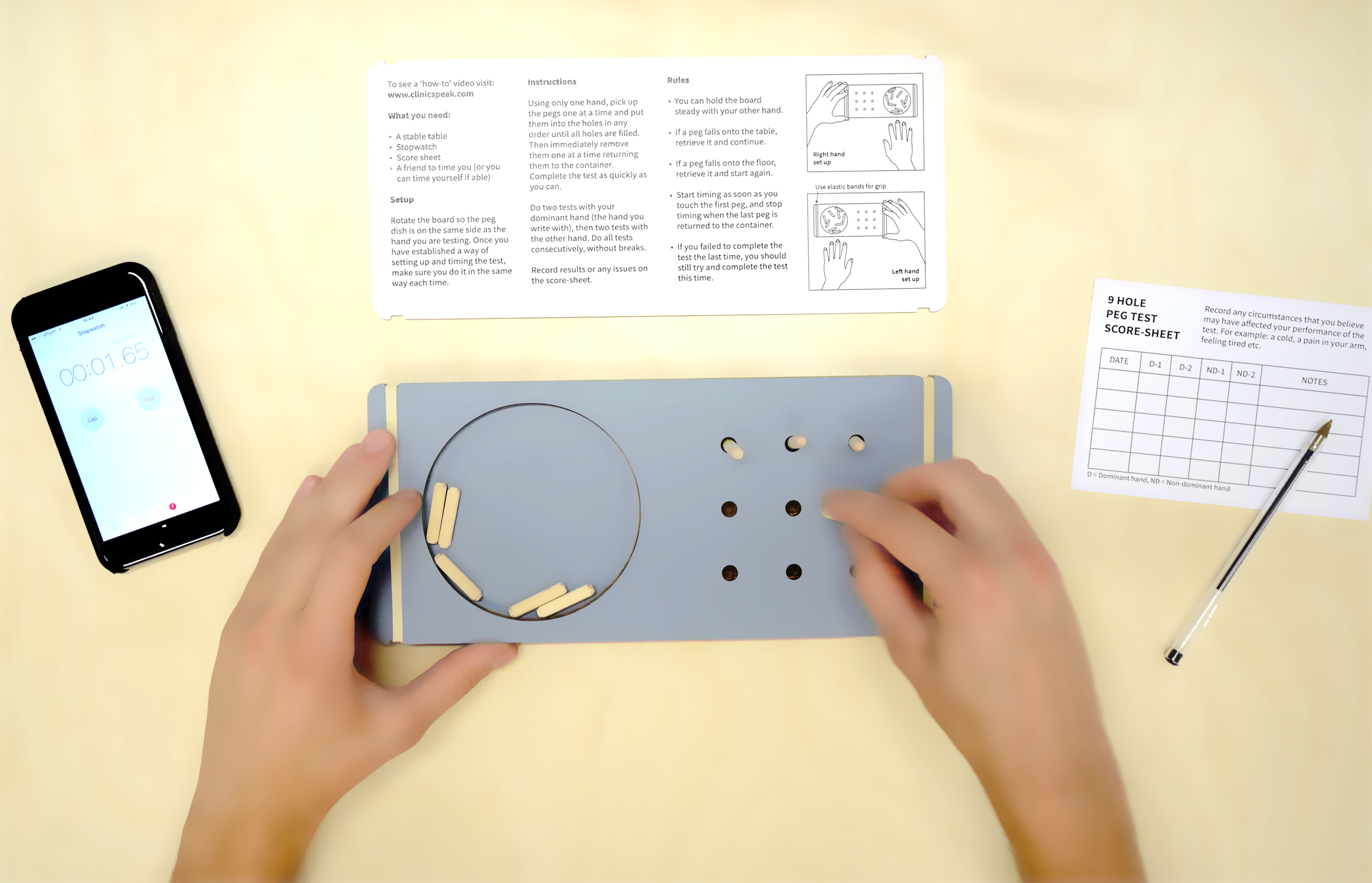
The cardboard 9 Hole Peg Test. Image credit: The Agency of Design

The 9HPT is a widely used tool for evaluating upper limb function and is a standard method in MS clinical trials [27, 28]. However, the standard 9HPT apparatus made of plastic is costly and the test usually administered by a clinician, limiting its access for patients to monitor their upper limb function themselves. To address these limitations, the ThinkHand campaign developed a low-cost cardboard version of the test (Fig. 4). This innovative measure was designed and produced by the campaign team, in collaboration with a design consultancy The Agency of Design, and was validated through an academic publication [16]. This low-cost and easily accessible cardboard 9HPT provides an alternative measure for upper limb function, making it more widely available to researchers and clinicians working in the field of MS. It was distributed to 12,000 delegates at the ECTRIMS conference and quickly became a success, receiving requests from around the world for its use in remote monitoring of hand function in patients and clinical and research teams.

## Co-design of a rehabilitation tool

**Fig. 5:**
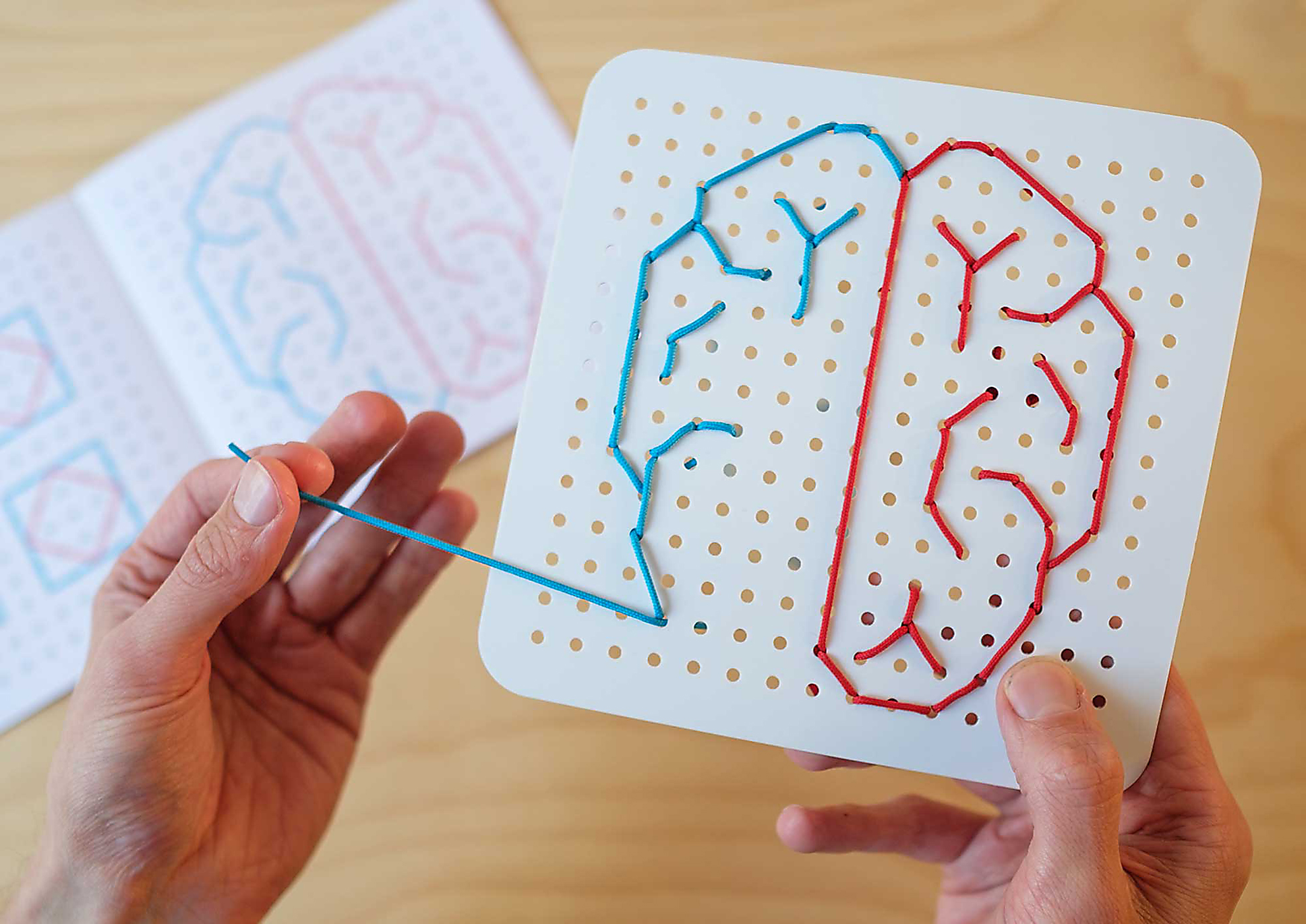
A photograph of the Under and Over rehabilitation tool. Image credit: The Agency of Design

After the international distribution and use of the cardboard 9HPT, the team received feedback that patients were using the tool more regularly than intended. Some were using it multiple times a week to see if they could improve on their time, ultimately using it like a rehabilitation tool. This supported other patient feedback the team had received from earlier qualitative work, where people with MS used everyday activities and objects to monitor changes in their hand and arm function at home. Anecdotes of using the ability to scrape a yoghurt pot clean every morning at breakfast to varying degrees of success would tell one person what their hand and arm function was like that day. This became a design-led exploration of alternative ways to measure, record and account for people’s experiences of change in hand and arm function in everyday life. Key to this design process was reviewing existing upper limb rehabilitation tools for both MS and other conditions, then including insights from patients about how they engaged with the c9HPT tool at home (Fig. 5).

The resulting tool, Under & Over, is a plastic board with two strings that people can thread to create a range of patterns. This tool was formally assessed in a remote study with a 12-week rehabilitation programme. The study found that a small, engaged, and motivated group of individuals with advanced MS were able to complete a remote rehabilitation program [17]. The study was the first fully remote study examining the effect of a targeted upper limb rehabilitation tool and demonstrates the feasibility and acceptability of delivering a remote program to individuals with advanced MS.

## Campaign success

The ThinkHand campaign contributed to several notable outcomes in the field of MS research and management. The campaign received financial support from private donors, commercial and government agencies which allocated resources towards the study of upper limb function in people with MS. Our activities contributed to the development of two clinical trials with emphasis on people with more advanced MS with upper limb function measured using the nine-hole-peg test as their primary outcome measures. (i) An international trial to assess ocrelizumab in people with primary progressive MS (O’HAND Study, NCT04035005), of which one of the co-authors (Giovannoni) became the principal investigator. (ii) A UK-wide trial called ChariotMS (NCT04695080), which focusses entirely on pwMS who are wheelchair users. From its inception, ChariotMS has been co-created and managed with PPIE input. The trial, testing cladribine tablets in pwMS with an EDSS between 6.5 and 8.5 is being supported by three charities (MS Society UK, National MS Society US, Barts Charity), the National Institute for Health and Care Research (NIHR) EME-Programme, and an industry partner (Merck Healthcare). If successful, both ChariotMS and O’HAND could lead to the first MS drug(s) licensed to protect upper limb function, including in pwMS who have so far been completely excluded from participating in MS DMT trials. With two trials underway that were facilitated by our campaign, the team won in 2019 the Influence Award in the public engagement award ceremony of the QMUL Centre for Public Engagement.

While the ThinkHand campaign aimed to address an important unmet need in the MS community, some pwMS expressed resistance to being regularly reminded of the limitations imposed by their condition. Similarly, some researchers and professionals in the field of MS found the campaign’s approach to be challenging to established perspectives and dogmas. This type of resistance to new ideas and perspectives is not uncommon in any field of research, as new ideas often challenge established views and can take time to gain acceptance [29]. Despite the slightly mixed reception, the ThinkHand campaign has had a lasting impact on the field of MS research and management and has provided patients with an innovative and independent method for monitoring their upper limb function.

### Learnings for practice

Through the experience of working with people with MS in creative practices of engagement and involvement, valuable insights emerge, offering significant learnings for practice that enable researchers to effectively tackle complex problems within clinical and research settings in new ways.

### Interdisciplinary collaboration

The campaign was an interdisciplinary collaboration, harnessing the collective expertise of multiple fields to tackle the complex and multifaceted nature of the so-called wicked problem. For example, the project involved collaboration between experts in areas such as lived experience, medicine, clinical research, design research, and social sciences to address the challenge at hand. This interdisciplinary approach not only facilitated a holistic understanding of the issue but also promoted innovative and creative exploration. Unlike approaches confined to a single field, the campaign benefited from a range of accountabilities and was not bound by the constraints of any singular research methodology. This flexibility allowed the project to employ diverse methods from various disciplines, enabling a more creative and responsive process.

### Systems thinking

The project used systems thinking to understand the interconnectedness of different components of the problem and how they influence each other. This approach was used to identify the root causes of lack of change in the field and design interventions that address these holistically. The impact of this has long lasting benefits for people with MS. For example, the cardboard 9HPT tool addressed health access issues brought about during the Covid-19 pandemic where the Neurology service at The Royal London Hospital used it as a remote test to monitor patients when they were unable to see them in person [30, 31].

### Co-creation with multiple stakeholders

The project involved co-creation with stakeholders, including people with MS, funders, charities, regulators, health care professionals and researchers to ensure that interventions were relevant and feasible. The project used participatory methods, such as co-design, to gather input and build engagement from these stakeholders. For example, the project engaged with people with advanced MS to understand exactly how they generate information on their hand function at home and used this to develop the Under & Over tool that could align with their needs and values.

### Experimentation and iteration

The project used an experimental and iterative approach, testing and refining interventions based on reflections and feedback from multiple audiences. This approach allowed the project to learn from failures, improve upon successful ideas and approaches, and continuously adapt to changing circumstances. For example, the team kept a commentary of the campaign on the MS Research Blog, engaging readers on the development of the campaign and sharing problems, challenges and developments, such as the ChariotMS trial. The project did not follow a traditional research process. The team had a practical understanding of the problem that guided activities while responding to iterative feedback from people with MS. In this way, it was not restricted to a predetermined process that could have limited aspects of its responsiveness.

## Conclusion

In summary, we have described the innovative approach undertaken by our team in the ThinkHand campaign, addressing the complex issue of upper limb function in pwMS. By actively involving patients in the process, the campaign not only raised awareness about the importance of upper limb function in MS but also contributed to the development of innovative ways to measure, record, and account for the change in hand and arm function in the everyday lives of people with MS. This has had a long-lasting influence on the field of MS research and management.

Moreover, the campaign underscores the immense potential of a patient-centred approach when grappling with complex issues in health research. By fostering openness, transparency, and honest discussions, patients were empowered to contribute and influence the trajectory of the project actively. This powerful impact of sustained patient engagement and involvement practices cannot be overstated.

The campaign’s realisation that traditional methods of communication and knowledge dissemination are inadequate for driving widespread change further emphasises the crucial lessons learned from involvement and engagement. Through these lessons, we recognise the necessity of working with more dynamic, engaging, and creative methods to comprehend the significance of this issue truly.

## Data Availability

No datasets were generated or analysed during the current study.

## Declarations

### Author information

AT is a design researcher and Senior Lecturer in PPIE at Queen Mary University of London. AT established and leads the Barts MS Patient Advisory Group and was the PI of the Under & Over study.

TB is an MS patient advocate and thought leader who provided ongoing support and advice as an ambassador for the ThinkHand campaign, particularly around engagement with the MS community.

CC lives with MS and is an Honorary Research Fellow (clinical trials patient communications) at the Blizard Institute.

RH, PB, MH, EC and BB live with MS and contributed as members of the Barts MS Patient Advisory Group to the development of ThinkHand activities and writing of this manuscript.

AS is a research neuro-physiotherapist at Barts Health NHS Trust and Honorary Clinical Lecturer at Queen Mary University of London and was involved in the Under & Over study.

KS is a Professor of Neurology and consultant neurologist. He is a Deputy Director of Clinical Research at QMUL & Barts Health NHS Trust, the Neurology Research lead, and the clinical lead of BartsMS. KS is the chief investigator of ChariotMS.

DB is a Professor of Immunology at Queen Mary University of London and is a frequent author of The MS Research Blog.

GG is a Professor of Neurology and MS consultant neurologist and runs the MS Selfie website.

## Abbreviations

QMUL: Queen Mary University of London
PPI: patient public involvement
DMTs: disease-modifying treatments
MS: Multiple Sclerosis
9HPT: Nine-Hole Peg Test
c9HPT: Cardboard Nine-Hole Peg Test
ECTRIMS: European Committee for Treatment and Research in Multiple Sclerosis
